# Infant Neural Sensitivity to Dynamic Eye Gaze Is Associated with Later Emerging Autism

**DOI:** 10.1016/j.cub.2011.12.056

**Published:** 2012-02-21

**Authors:** Mayada Elsabbagh, Evelyne Mercure, Kristelle Hudry, Susie Chandler, Greg Pasco, Tony Charman, Andrew Pickles, Simon Baron-Cohen, Patrick Bolton, Mark H. Johnson, the BASIS Team

**Affiliations:** 1 Centre for Brain and Cognitive Development, Birkbeck College, University of London, London WC1E 7HX, UK; 2 Department of Psychiatry, McGill University, Montreal, Quebec H3A 1A1, Canada; 3 Olga Tennison Autism Research Centre, School of Psychological Science, La Trobe University, Bundoora, Victoria 3086, Australia; 4 Centre for Research in Autism and Education, Institute of Education, University of London, London WC1H 0AL, UK; 5 Institute of Psychiatry, King's College London, London SE5 8AF, UK; 6 Autism Research Centre, University of Cambridge, Cambridge CB2 8AH, UK

## Abstract

Autism spectrum disorders (henceforth autism) are diagnosed in around 1% of the population [1]. Familial liability confers risk for a broad spectrum of difficulties including the broader autism phenotype (BAP) [2, 3]. There are currently no reliable predictors of autism in infancy, but characteristic behaviors emerge during the second year, enabling diagnosis after this age [4, 5]. Because indicators of brain functioning may be sensitive predictors, and atypical eye contact is characteristic of the syndrome [6–9] and the BAP [10, 11], we examined whether neural sensitivity to eye gaze during infancy is associated with later autism outcomes [12, 13]. We undertook a prospective longitudinal study of infants with and without familial risk for autism. At 6–10 months, we recorded infants' event-related potentials (ERPs) in response to viewing faces with eye gaze directed toward versus away from the infant [14]. Longitudinal analyses showed that characteristics of ERP components evoked in response to dynamic eye gaze shifts during infancy were associated with autism diagnosed at 36 months. ERP responses to eye gaze may help characterize developmental processes that lead to later emerging autism. Findings also elucidate the mechanisms driving the development of the social brain in infancy.

## Highlights

► Family risk for autism confers subtle differences in brain function in infants ► Atypical ERPs in infants when viewing eye gaze data associates with later autism diagnosis ► Robust prediction of autism will require an understanding of risk and protective factors

## Results and Discussion

In the present study, we ascertained whether measurements of specific brain function might be sensitive predictors of later outcomes in infants at familial risk for autism spectrum disorders, a condition currently defined on the basis of qualitative impairments in social skills and communication and the presence of rigid, stereotyped, and repetitive behaviors. We tested the hypothesis that atypical neural responses to eye gaze in infants at familial risk for autism relate to emerging symptoms of autism assessed behaviorally at 3 years of age. We followed up a group of 104 infants: 54 at risk for autism and 50 control infants who had no family history of autism from the age of 6–10 months and through to 36 months.

**Figure 1:**
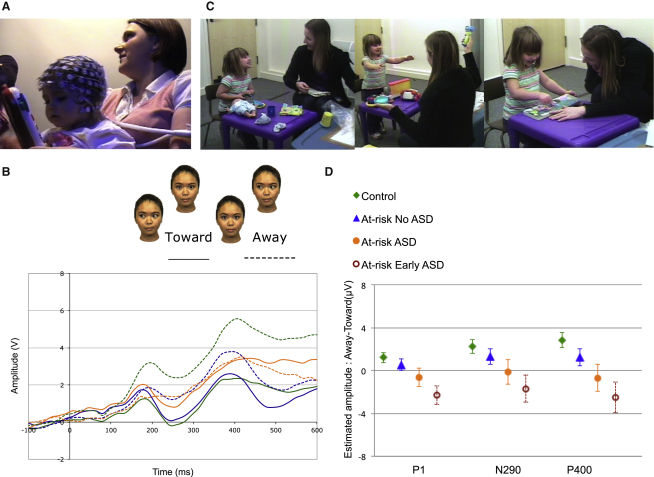
Association between Infant ERPs in Response to Eye Gaze and Autism Outcomes (A) Participating families first visited the lab when their infants were 6–10 months of age. Electrophysiological recording was done during this visit. Infants were prepared for the EEG session. (B) Electrophysiological response to gaze shifts over occipitotemporal channels. (C) Around 2 and 3 years of age, the same infants were tested by an independent team using several measures including the ADOS, a semistructured observational measure of autism-related characteristics. Based on information from all visits, combined with expert clinical judgment, infants in the at-risk group were classified as having ASD or not. (D) Controlling for age at the first visit, significant condition × risk-group interactions were observed for the amplitude of the P400 [*F*(1,92) = 6.7, p = 0.01]; planned post hoc tests focused on within-group difference between response to direct versus averted gaze controlling for age at baseline and developmental level at 36 months. Estimated mean differences between responses to gaze toward versus away are displayed for each group (standard error bars are displayed). Findings suggest that differentiation between gaze toward versus away was reliable in the both the control group (p < 0.001) and the at-risk without ASD group (p = 0.04). By contrast, the at-risk group that developed ASD showed no differentiation (p = 0.67) nor did the subgroup that developed early and persistent symptoms (p = 0.27). Findings from static face and face versus noise contrasts are presented in Figure S1 and Table S1.

At 6–10 months of age, we recorded infants' event-related potentials (ERPs) using a 128-channel hydrocel infant net, in response to dynamic gaze shifts toward versus away from the infant (Figure 1). In addition to this primary contrast, we also examined the extent to which any observed patterns extend to more general face-processing mechanisms, by measuring ERPs in response to (1) the first static presentations of faces appearing in the gaze-shift sequence displaying direct versus averted gaze (previously used in our preliminary study, [10]) and (2) face versus visual noise (the latter was constructed from the same face stimuli, with randomization of the phase spectra while keeping constant the amplitude and color spectra [15]). We measured characteristics of three infant components, time-locked to the onset of stimulus presentation. The P1, N290, and the P400 components, quantified by their amplitude or latency, are modulated in a number of face-perception tasks, including tests of sensitivity to the direction of eye gaze in infants as young as four months [16]. Relevant experimental findings from different laboratories, including our own, have identified these ERP components in infants as precursors of the well-established face-sensitive N170 component in adults [17]. In interpreting our results, we did not discriminate between amplitude or latency changes, because both measures equally reflect differences in the neural response to the contrasts of interest.

We constrained our general linear model analyses (Figure 1, detailed in Figure S1 and Table S1 available online) against type 1 error in three ways. First, we tested hypothesis-driven within-group effects only when higher order interactions were significant (using Greenhouse-Geisser corrections; Table S1). Second, we focused our primary analysis on the dynamic gaze-shift stimuli (Figure 1) because these are relevant to naturalistic shared attention contexts known to be disrupted in autism [12, 13]. Furthermore, several ERP studies have suggested that, relative to static face presentation, dynamic shifts engage a wider range of social brain mechanisms [18, 19]. Finally, in view of our findings from our preliminary study [10], we were specifically interested in the P400 response relative to the P1 and N290. Full results of other components and contrasts are presented in Figure S1 and Table S1.

### Face-Related ERPs Distinguish Infants at Risk from the Control Group

Our results generally replicated and extended previous reports, differentiating infants at risk from controls with no family history of autism [10, 20–22]. In the present study, a significant risk group × condition interaction was observed for the P400 response in response to dynamic gaze shifts toward versus away from the infant. Notably, in the dynamic gaze condition the control group, but not the group of infants at risk, showed a significant difference in response to gaze toward, compared to away from, the infant (Figure 1; within group contrasts: control group p < 0.001; combined at-risk group p = 0.48). However, these risk-group effects were not restricted to dynamic gaze contrasts but appear to extend to other face processing mechanisms, i.e., static direct vs. averted gaze and face vs. noise (Figure S1; Table S1).

Overt behavioral signs of autism are rarely observable in the first year. Nevertheless, our results support the conclusion that cognitive and brain function measures can successfully differentiate groups of infants at risk from low-risk control within the first year of life [4]. Consistent with our current findings, these effects have been reported in visual processing [20] and in flexibility of switching attention [21]. Direct measurement of brain activity has also revealed early risk-group differences in response to face stimuli [22] and in sensitivity to the direction of eye gaze [10]. Our findings, from a larger sample than those reported previously, verified that such group effects are observable within the first year of life, across both face and gaze processing mechanisms. Within this early period, risk for autism appears to confer a range of differences in the developing brain.

### Infant Brain Response to Dynamic Gaze Is Associated with Autism at 36 Months

In order to determine how these infancy findings are associated with later behavioral outcomes, an independent team conducted clinical research assessments of the same infants when they reached 24 months and 36 months of age, with an outcome diagnosis of Autism spectrum disorders (ASD) at 36 months for 17 toddlers from the at-risk group. Testing the relationship between infant brain function measures and later outcomes followed on from the previous analysis based on risk groups but involved splitting the at-risk group based on their outcomes (control, at-risk ASD, at-risk no ASD). Post hoc tests focused on within group differences controlling for overall developmental level to ensure specificity of any observed effects to the diagnostic group. The focus on within-group differences ensured that any unexpected global differences in baseline electroencephalography (EEG) would not drive results of the ERP post hoc tests. As infants, the control group, as well as the at-risk group with no ASD, showed a higher P400 amplitude for gaze shifts away versus toward, whereas the ASD group did not differentiate the two conditions (Figure 1D). The face versus noise contrast did not distinguish the at-risk ASD group from the two other groups, suggesting some degree of specificity of the effect to dynamic gaze-shift condition (Figure S1, Table S1).

Given variability in developmental pathways leading to diagnosis at 3 years, we were interested in examining whether these early brain differences may relate more specifically to social and communication impairment emerging during the second year and then persisting into the third year (“early and persistent ASD”). This question is also important in view of previous studies on typical development suggesting that infant sensitivity to eye gaze relates to social and communication skills emerging around the second birthday. Those skills were assessed at 2 and 3 years of age using the Autism Diagnostic Observation Schedule (ADOS; Figure 1C; [23]). Around 60% of infants in the ASD group exhibited clear symptoms when assessed at the age of two, meeting ADOS clinical cutoff at that age. We examined the P400 response to gaze shift (toward versus away) after removing from the ASD group those infants who did not meet ADOS clinical cut-off criteria at the age of 2, leaving nine infants in the “early and persistent ASD” sample. The same lack of differentiation between gaze toward and away was observed in this subgroup (p = 0.27). Moreover, the early and persistent subgroup also showed differences in other gaze-related ERP components, namely the P1 and N290 (Figure S1; Table S1). This finding suggests that ERP responses to dynamic gaze shifts may help characterize distinct developmental profiles leading to autism.

Neither static gaze nor face versus noise contrasts reliably distinguished the ASD group from the two other groups. Notwithstanding this pattern, findings from the static gaze condition (Figure S1), suggest that latency of the P400 in response to direct versus averted gaze did not differ in the control group (p = 0.89) or at-risk group who developed ASD (p = 0.54), but that within the at-risk group who did not develop ASD the response to direct gaze was slower than to averted gaze (p = 0.007). Given the heterogeneity of outcomes in the group that did not develop autism, we further verified that this within-group difference was not driven by those infants who had other forms of developmental concerns not meeting clinical thresholds for an autism diagnosis. We confirmed that the effect was primarily driven by those infants at risk who were found to be developmentally typical at 3 years of age (p = 0.04) and less reliably in the group with other developmental concerns (p = 0.09). This pattern suggests that at least some risk-group differences may be driven by infants who do not go on to develop autism in toddlerhood.

### Are Brain Function Effects Driven by Differences in Looking Behavior?

It has been frequently suggested that individuals with autism [24] and their nonaffected relatives [11] exhibit differences in the scanning of faces and social scenes. Such putative scanning differences in the period of infancy may explain or modulate the neural response to eye gaze observed in our study. However, in general, evidence from infants at risk has thus far indicated typical patterns of scanning of social scenes within the first year of life, supporting the view that atypical scanning emerges over the early developmental period [4]. In fact, one study reported that normative face-scanning patterns modulate later language outcomes in both infants at risk for autism and control groups [25]. Nevertheless, we considered it important to ascertain whether such differences were present in the current study. Among a number of different methodological controls we employed (detailed in the Supplemental Experimental Procedures), we used a separate eye tracking task to examine the amount of time spent looking at the eye region of the face. This allowed us to assess whether the differences arising in the neural response to eye gaze could be attributed to decreased scanning of, or attention to, the eye region.

Eye-tracking data from 93 of the 104 infants were retained for analysis if at least 1.5 s of looking time were accumulated across one or both trials. Rectangular Areas of Interest (AOIs) were defined around the eye region, the mouth region, and other areas covering the remaining non-face regions. No differences were observed across the risk-groups or based on outcome groups in the percentage of time spent fixating on the models' eyes relative to other regions (Table 1). These findings confirm that brain function differences observed in the group of infants at risk are not driven by overt differences in visual scanning of social scenes. These findings are consistent with the emerging body of evidence suggesting that the expression of risk for autism within the first year is subtle when measured using overt behavioral markers [4].

### Ontogeny of Autism and Its Broader Phenotype

Typical infants' sensitivity to eye gaze in the first year of life is a precursor to a range of social-communicative skills that emerge as early as 18 months. Very early sensitivity to eye gaze [26, 27] develops rapidly and takes on the more sophisticated form of joint attention, where the infant shares the adult's focus of interest. The infant can then use this social context to develop a wide array of skills (e.g., to learn words, interpret facial expressions, and understand intentions). This evidence has led to the proposition that, in autism, disruptions in the early instantiation of brain networks underlying social perception, results in decreased attention to, or reduced interest in, the social world [12, 13]. This early perturbation, potentially affecting sensitivity to eye gaze, interferes with the emergence of typical developmental milestones. The cascading effects eventually derail the development of social cognition.

Although converging evidence from behavioral [6, 7] and neuroimaging [8, 9] studies supports the notion that atypical sensitivity to eye gaze is characteristic of individuals with autism and their unaffected relatives [11], our study is the first to formally test the aforementioned developmental account. Our findings, however, suggest that atypical brain function precedes the onset of overt behavioral signs and subsequently symptoms. Although overt behavioral signs of autism are rarely observable in the first year, response to dynamic gaze shifts during the first year of life distinguished the group of infants who later developed autism. The same pattern of findings was not seen with a much more salient stimulus difference (face versus visual noise stimuli), suggesting at least some degree of effect specificity. This pattern is consistent with the view that whereas sensitivity to static gaze information emerges early in development, dynamic gaze shifts are likely to engage multiple bottom-up and top-down social brain networks, including those concerned with prediction of future events [28]. Thus, the dynamic nature of our gaze-shift stimuli involving rapid changes may be a critical feature of the task we used, and future research needs to determine in more detail the specificity or otherwise of these effects to eye gaze.

Our findings, from a larger sample than those reported previously, also verified that risk group effects are observable within the first year of life, across both face and gaze processing mechanisms. Within this early period, risk for autism appears to confer a range of differences in the developing brain. Future work will also be required to ascertain why the secondary stimulus contrasts (static direct versus averted gaze and face versus visual noise) revealed risk-group differences that were not due to the subset of infants who go on to a later diagnosis of autism. One possibility is that early brain function differences reflect early manifestations of the broader autism phenotype that will become more clearly evident in behavior later in life. Another possibility is that at least some early differences may reflect protective factors or mechanisms of brain adaptation in those infants at risk who go on to exhibit a typical behavioral repertoire, a pattern previously referred to as canalization [4]. Preliminary evidence in the current study related to the response to static direct versus averted gaze appears to support this possibility, consistent with suggestions from studies of older siblings of individuals diagnosed with autism [29, 30].

Taken together, our findings potentially allow for the early identification of those infant siblings who are at highest risk for developing later impairments, paving the way for the more selective targeting of early intervention efforts and procedures. In the future, more reliable diagnostic and, more importantly, prognostic indicators of the condition may become clear through a better understanding of the way in which very early differences in the brain functioning of infants at risk relate to variable developmental pathways [4]. Previous attempts to draw conclusions regarding clinical utility of laboratory measures in adults and infants alike have demonstrated the need for caution [31], not least because although group mean differences emerge, there is still considerable overlap between groups in individual infant responses. Whereas clinical utility can only be established using much larger and unselected population samples, studies like ours can offer important clues regarding the correspondence between infant early laboratory measures and later clinical outcomes. More robust prediction of clinical diagnosis may require a combination of a number of risk and protective factors, including response to gaze.

## Experimental Procedures

### Participants and Clinical Characterization

Recruitment, ethical approval (UK National Health Service National Research Ethics Service London REC 08/H0718/76), and informed consent, as well as background data on participating families, were made available for the current study through the British Autism Study of Infant Siblings (BASIS), a UK collaborative network facilitating research with infants at risk for autism (www.basisnetwork.org). Families enroll from various regions when their babies are younger than 5 months of age, and they are invited to attend multiple research visits until their children reach 3 years of age or beyond. Each visit lasts a day or two and is adapted to meet the families' needs. Measures collected are anonymized and shared among scientists to maximize collaborative value and to minimize burden on the families. A clinical advisory team of senior consultants works closely together with the research team(s) and, if necessary, with the family's local health services, to ensure that any concerns about the child arising during the study are adequately addressed.

One hundred and four infants from BASIS (independent from the pilot group [10]) took part in the current study (54 at risk, and 50 control). Twenty-one of the at-risk infants were male and 33 were female. Twenty-one of the low-risk infants were male and 29 were female. Along with several other measures, the infants were seen for the ERP task at the Centre for Brain and Cognitive Development when they were 6–10 months of age (mean = 238.3 days, SD = 37.2). Subsequently, 52 (from 54) of those at risk for ASD were seen for assessment around the second birthday (mean = 23.9 months, SD = 1.2) and 53 around their third birthday (mean = 37.7 months, SD = 3.0), by an independent team at the Centre for Research in Autism and Education, Institute of Education.

During the 36 month visit, a battery of clinical research measures was administered including the Autism Diagnostic Observation Schedule and the Autism Diagnostic Interview. Consensus ICD-10 criteria were used to ascertain diagnosis in a subgroup of infants at risk using all available information from all visits by experienced researchers (T.C., K.H., S.C., G.P). The Supplemental Experimental Procedures present detailed participant characteristics including ascertainment of risk status, background measures at each visit, and outcome characterization including clinical classification.

### ERP Task at 6–10 Months

In our previously published preliminary work [10], we investigated group differences between infants at risk for autism and low-risk controls, aged 10 months in their response to static direct versus averted gaze (for details on the pilot study see Supplemental Experimental Procedures). For the current study, a modified ERP task was administered during the first visit.

The infants sat on their parents' laps at a 60 cm distance from a 40 × 29 cm computer screen. Gaze during stimulus presentation was recorded by video camera. Each trial block began with a static colorful fixation stimulus followed by a color image of one of four female faces, with gaze directed either toward or away from the infant (Figure 1). In subsequent trials of the same block, the face remained on the screen but displayed three to six gaze shifts, alternating from directed toward to away from the infant. Faces were aligned with the center of the screen with the eyes appearing at the same location as the fixation stimuli, to ensure that infants were fixating the eye region. The faces subtended 21 × 14 degrees of visual angle. In addition to face trial blocks, during approximately one third of all blocks, infants were presented with “visual noise” stimuli. The latter were constructed from the same faces presented within the task, by randomizing the phase spectra while keeping the amplitude and color spectra constant [15]. Fixation stimuli, preceding the onset of the face and noise stimuli, subtended approximately 1.6 × 1.6 degrees and were presented for a variable duration of 800 to 1,200 ms. Each trial lasted for 1,000 ms.

A 128 channel Hydrocel Sensor Net was mounted on each infant's head, while they were seated on the parent's lap in front of the stimulus screen. When the infant was attending toward the screen, trials were presented continuously for as long as the infant remained attentive, with brain electrical activity measured simultaneously using the vertex as a reference (Cz in the conventional 10/20 system). EGI NetAmps 200 was used (gain = 1,000). Data were digitized with a sampling rate of 500 Hz and band-pass filtered between 0.1–100 Hz. Subsequent to artifact rejection, ERPs were ascertained based on visual inspection of grand averages (detailed in Supplemental Experimental Procedures).

### Supporting Eye-Tracking Task at 6–10 Months

During their first visit, infants were administered a battery of eye-tracking tasks several hours before the ERP task was undertaken and containing no identical stimuli. Of particular relevance for the current study was a condition in which infants were presented with videos of female faces displaying gaze shifts toward or away from the infant. Looking behavior was recorded with a Tobii eye tracker. The Tobii system has an infrared light source and a camera mounted below a 17 inch flat-screen monitor to record corneal reflection data. Gaze direction of each eye is measured separately, and from these measurements, the Tobii system evaluates where on the screen the individual is looking. During the task, the infant was seated on the parent's lap, at 50–55 cm from the Tobii screen, with height and distance of the screen adjusted to obtain good tracking of the eyes. A five-point calibration sequence was run, and recording and presentation of the study stimuli only started when at least four calibration points were marked as properly attuned to each eye. Gaze data were recorded at 50 Hz.

Each infant was presented with two video sequences with different female faces. Each began with an animated fixation stimulus attracting the infant's attention to the center of the screen. Once fixated, a still face was presented for 5 s. Subsequently, the model shifted her gaze away from the infant and then alternated gaze shifts back toward and again away from the infant (turning left or right in pseudorandom order) for a maximum of ten repetitions.

## Figures and Tables

**Table 1:** Supporting Eye-Tracking Task

	Control		At-risk					
	mean	SD	Combined		No ASD		ASD	
			mean	SD	mean	SD	mean	SD
n	45		40		27		13	
Total looking time (sec)	7.7	*3.3*	8.0	*3.3*	7.3	*3.2*	9.4	*3.0*
% eye	65.2	*20.3*	72.2	*23.1*	74.7	*17.6*	67.2	*32.1*
*p*	*Control versus at-risk = 0.14*				*No ASD versus ASD = 0.34*			
% Mouth	23	*20.2*	17	*16.4*	15.9	*13.7*	20.7	*21.3*
*p*	*Control versus at-risk = 0.21*				*No ASD versus ASD = 0.39*			

This table shows number of infants, average amount of looking time on each trial, and distribution of gaze across different areas of interest. P values yielded from the pairwise comparison of risk group or outcome subgroup means.

## Supplemental Information

Document S1. Figures S1–S3, Tables S1–S3, and Supplemental Experimental Procedures

## References

[1] Baird G., Simonoff E., Pickles A., Chandler S., Loucas T., Meldrum D., Charman T. (2006). Prevalence of disorders of the autism spectrum in a population cohort of children in South Thames: the Special Needs and Autism Project (SNAP). Lancet.

[2] Pickles A., Starr E., Kazak S., Bolton P., Papanikolaou K., Bailey A., Goodman R., Rutter M. (2000). Variable expression of the autism broader phenotype: findings from extended pedigrees. J. Child Psychol. Psychiatry.

[3] Losh M., Adolphs R., Poe M.D., Couture S., Penn D., Baranek G.T., Piven J. (2009). Neuropsychological profile of autism and the broad autism phenotype. Arch. Gen. Psychiatry.

[4] Elsabbagh M., Johnson M.H. (2010). Getting answers from babies about autism. Trends Cogn. Sci. (Regul. Ed.).

[5] Zwaigenbaum L., Thurm A., Stone W., Baranek G., Bryson S., Iverson J., Kau A., Klin A., Lord C., Landa R. (2007). Studying the emergence of autism spectrum disorders in high-risk infants: methodological and practical issues. J. Autism Dev. Disord..

[6] Baron-Cohen S., Baldwin D.A., Crowson M. (1997). Do children with autism use the speaker's direction of gaze strategy to crack the code of language?. Child Dev..

[7] Leekam S.R., Hunnisett E., Moore C. (1998). Targets and cues: gaze-following in children with autism. J. Child Psychol. Psychiatry.

[8] Grice S.J., Halit H., Farroni T., Baron-Cohen S., Bolton P., Johnson M.H. (2005). Neural correlates of eye-gaze detection in young children with autism. Cortex.

[9] Senju A., Tojo Y., Yaguchi K., Hasegawa T. (2005). Deviant gaze processing in children with autism: an ERP study. Neuropsychologia.

[10] Elsabbagh M., Volein A., Csibra G., Holmboe K., Garwood H., Tucker L., Krljes S., Baron-Cohen S., Bolton P., Charman T. (2009). Neural correlates of eye gaze processing in the infant broader autism phenotype. Biol. Psychiatry.

[11] Dalton K.M., Nacewicz B.M., Alexander A.L., Davidson R.J. (2007). Gaze-fixation, brain activation, and amygdala volume in unaffected siblings of individuals with autism. Biol. Psychiatry.

[12] Dawson G., Webb S., Schellenberg G.D., Dager S., Friedman S., Aylward E., Richards T. (2002). Defining the broader phenotype of autism: genetic, brain, and behavioral perspectives. Dev. Psychopathol..

[13] Johnson M.H., Griffin R., Csibra G., Halit H., Farroni T., de Haan M., Tucker L.A., Baron-Cohen S., Richards J. (2005). The emergence of the social brain network: evidence from typical and atypical development. Dev. Psychopathol..

[14] Senju A., Johnson M.H. (2009). The eye contact effect: mechanisms and development. Trends Cogn. Sci. (Regul. Ed.).

[15] Halit H., Csibra G., Volein A., Johnson M.H. (2004). Face-sensitive cortical processing in early infancy. J. Child Psychol. Psychiatry.

[16] de Haan M., Johnson M.H., Halit H. (2003). Development of face-sensitive event-related potentials during infancy: a review. Int. J. Psychophysiol..

[17] Bentin S., McCarthy G., Perez E., Puce A., Allison T. (1996). Electrophysiological studies of face perception in humans. Journal of Cognitive Neuroscience.

[18] Conty L., N'Diaye K., Tijus C., George N. (2007). When eye creates the contact! ERP evidence for early dissociation between direct and averted gaze motion processing. Neuropsychologia.

[19] Puce A., Allison T., Bentin S., Gore J.C., McCarthy G. (1998). Temporal cortex activation in humans viewing eye and mouth movements. J. Neurosci..

[20] McCleery J.P., Allman E., Carver L.J., Dobkins K.R. (2007). Abnormal magnocellular pathway visual processing in infants at risk for autism. Biol. Psychiatry.

[21] Elsabbagh M., Volein A., Holmboe K., Tucker L., Csibra G., Baron-Cohen S., Bolton P., Charman T., Baird G., Johnson M.H. (2009). Visual orienting in the early broader autism phenotype: disengagement and facilitation. J. Child Psychol. Psychiatry.

[22] McCleery J.P., Akshoomoff N., Dobkins K.R., Carver L.J. (2009). Atypical face versus object processing and hemispheric asymmetries in 10-month-old infants at risk for autism. Biol. Psychiatry.

[23] Lord C., Risi S., Lambrecht L., Cook E.H., Leventhal B.L., DiLavore P.C., Pickles A., Rutter M. (2000). The autism diagnostic observation schedule-generic: a standard measure of social and communication deficits associated with the spectrum of autism. J. Autism Dev. Disord..

[24] Klin A., Lin D.J., Gorrindo P., Ramsay G., Jones W. (2009). Two-year-olds with autism orient to non-social contingencies rather than biological motion. Nature.

[25] Young G.S., Merin N., Rogers S.J., Ozonoff S. (2009). Gaze behavior and affect at 6 months: predicting clinical outcomes and language development in typically developing infants and infants at risk for autism. Dev. Sci..

[26] Farroni T., Csibra G., Simion F., Johnson M.H. (2002). Eye contact detection in humans from birth. Proc. Natl. Acad. Sci. USA.

[27] Senju A., Csibra G. (2008). Gaze following in human infants depends on communicative signals. Curr. Biol..

[28] Guiraud J.A., Kushnerenko E., Tomalski P., Davies K., Ribeiro H., Johnson M.H., BASIS Team (2011). Differential habituation to repeated sounds in infants at high risk for autism. Neuroreport.

[29] Kaiser M.D., Hudac C.M., Shultz S., Lee S.M., Cheung C., Berken A.M., Deen B., Pitskel N.B., Sugrue D.R., Voos A.C. (2010). Neural signatures of autism. Proc. Natl. Acad. Sci. USA.

[30] Belmonte M. (2010). Visual attention in autism families: ‘unaffected’ sibs share atypical frontal activation. J Child Psychol Psychiatry.

[31] Walsh P., Elsabbagh M., Bolton P., Singh I. (2011). In search of biomarkers for autism: scientific, social and ethical challenges. Nat. Rev. Neurosci..

