# Supplementary material for: Infant Neural Sensitivity to Dynamic Eye Gaze Is Associated with Later Emerging Autism
Source: Curr Biol. 2012 Feb 21;22(4):338–42. doi: 10.1016/j.cub.2011.12.056 (PMC3314921; doi:10.1016/j.cub.2011.12.056)
Supplement: Document S1. Figures S1–S3, Tables S1–S3, and Supplemental Experimental Procedures [file mmc1.pdf]

Current Biology, Volume *22*

Supplemental Information

Infant Neural Sensitivity to Dynamic Eye Gaze Is Associated with Later Emerging Autism

Mayada Elsabbagh, Evelyne Mercure, Kristelle Hudry, Susie Chandler, Greg Pasco, Tony Charman, Andrew Pickles, Simon Baron-Cohen, Patrick Bolton, Mark H. Johnson, and The BASIS Team

Supplemental Inventory

1. Supplemental Figures and Tables

Figure S1, related to Figure 1

Figure S2

Figure S3

Table S1, related to Figure 1

Table S2

Table S3

2. Supplemental Experimental Procedures

3. Supplemental References

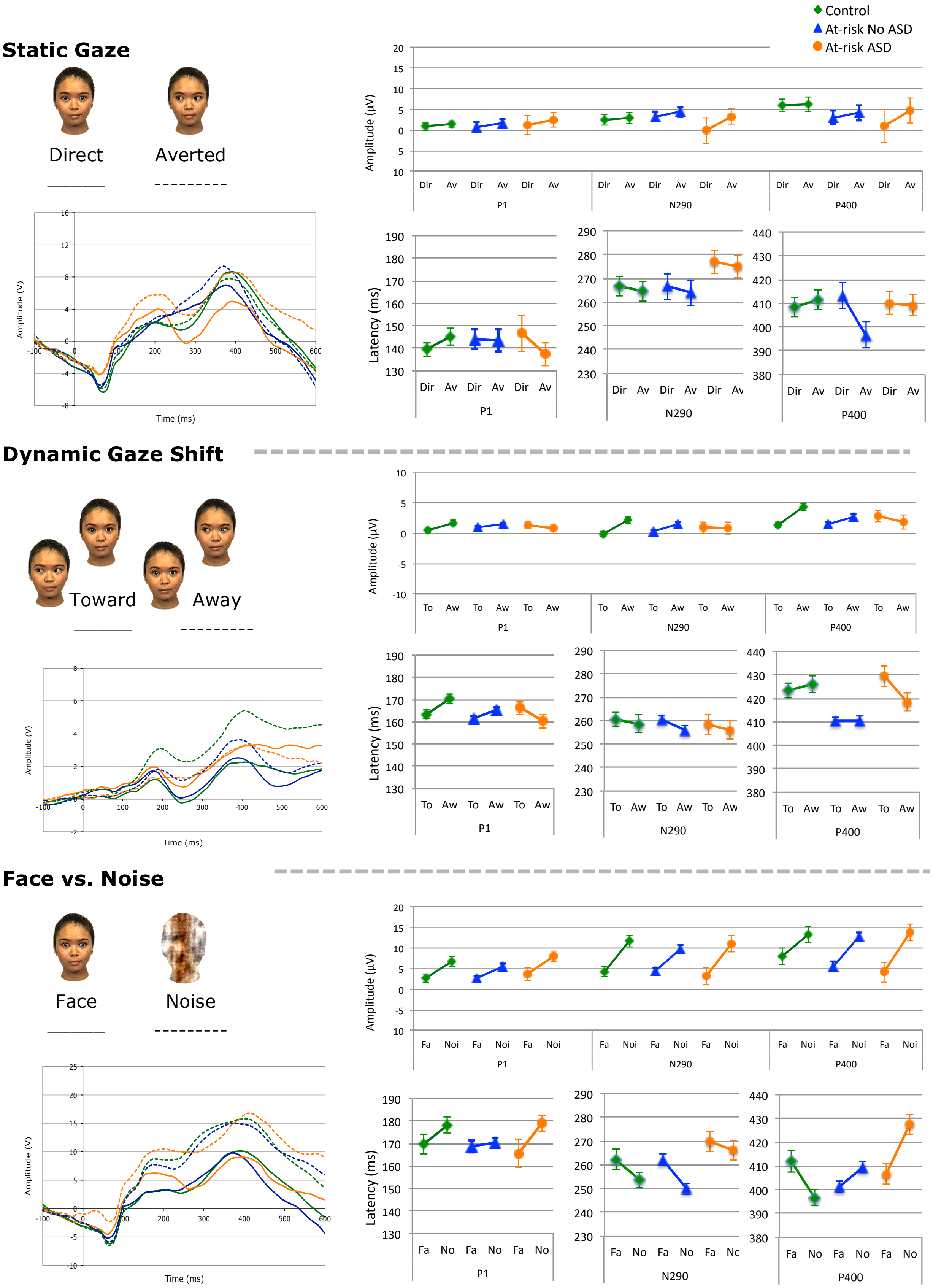
Static Gaze
Direct
Averted
Control
At-risk No ASD
At-risk ASD
Amplitude (μV)
Latency (ms)
Amplitude (V)
Time (ms)
Dir
Av
P1
N290
P400
Dynamic Gaze Shift
Toward
Away
To
Aw
Face vs. Noise
Face
Noise
Fa
Noi
No

Figure S1. Related to Figure 1*.*

Electrophysiological response over task-sensitive occipito-temporal channels (Left Panel), Means and standard errors of amplitude and latency of the three face-sensitive components in each group (Right Panel).

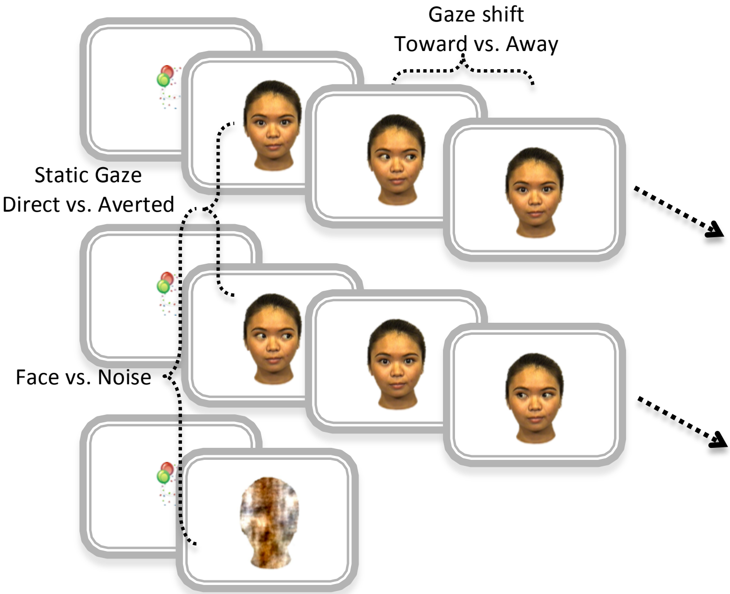
Gaze shift
Toward vs. Away
Static Gaze
Direct vs. Averted
Face vs. Noise

Figure S2.

ERP Task and Trial Grouping corresponding to the three contrasts: Static gaze, dynamic gaze, face vs. noise.

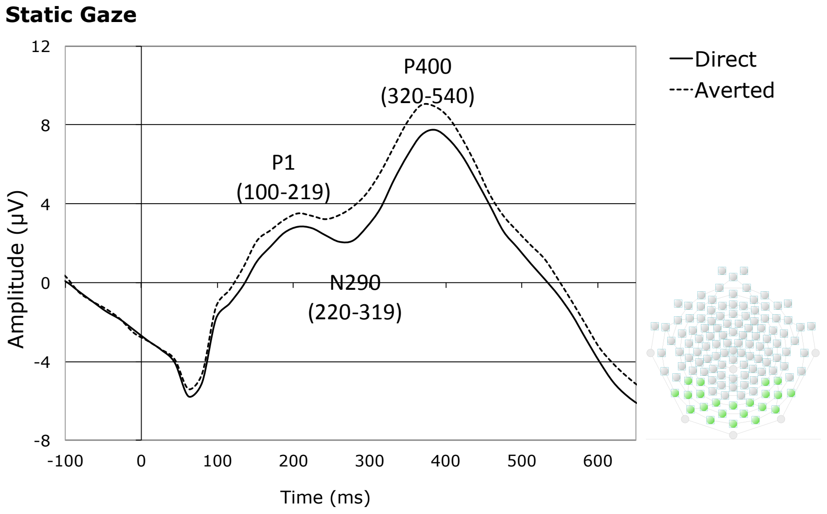
Static Gaze
Direct
Averted
P400
(320-540)
P1
(100-219)
N290
(220-319)
Amplitude (μV)
Time (ms)
12
8
4
0
-4
-8
-100
0
100
200
300
400
500
600

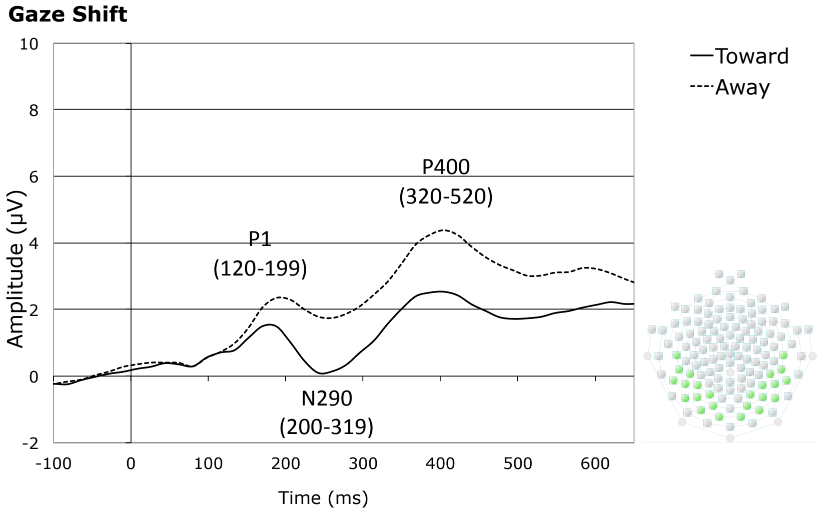
Gaze Shift
Toward
Away
P400
(320-520)
P1
(120-199)
N290
(200-319)
Amplitude (μV)
Time (ms)
10
8
6
4
2
0
-2
-100
0
100
200
300
400
500
600

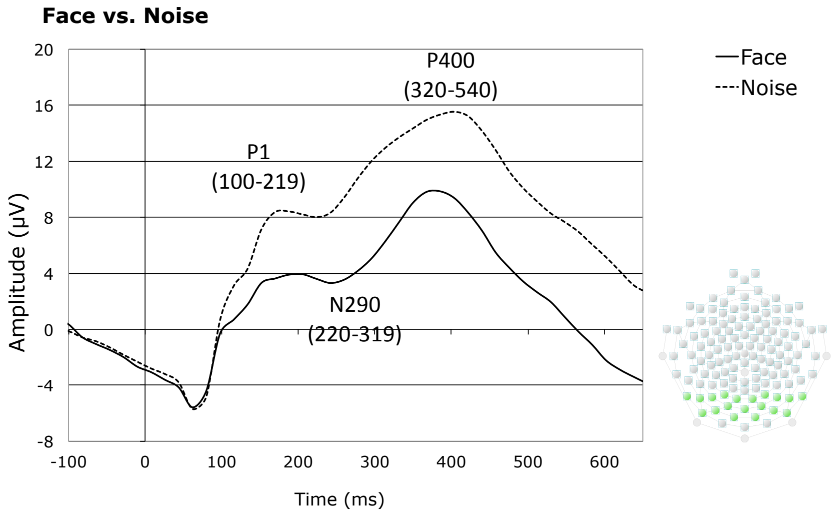
Face vs. Noise
Face
Noise
P400
(320-540)
P1
(100-219)
N290
(220-319)
Amplitude (μV)
Time (ms)
20
16
12
8
4
0
-4
-8
-100
0
100
200
300
400
500
600

Figure S3.

Average waveforms for all infants across the three contrasts over selected channels where the task-dependent characteristic waveform was observed. The three components P1, N290, and P400 are labelled along with the temporal window during which the peaks were defined.

Table S1. Related to Figure 1.

**A. Risk Group Effects (*p* values)**						
	P1		N290		P400	
	Amp	Lat	Amp	Lat	Amp	Lat
Static gaze condition x Risk group	.44	.22	.23	.86	.15	.02*
Ctl: Direct vs. Averted						.53
At-risk: Direct vs. Averted						.02*
Dynamic gaze condition x Risk group	.06	.02*	.09	.47	.01*	.49
Ctl: Toward vs. Away		.001*			<.001	
At-risk: Toward vs. Away		.77			.48	
Face condition x Risk group	.60	.84	.49	.89	.12	.02*
Ctl: Face vs. Noise						.09*
At-risk: Face vs. Noise						.07*
**B. Outcome group effects (*p* values)**						
Static gaze condition x outcome	.92	.49	.58	.99	.19	.04*
ASD: Direct vs. Averted						.54
No ASD: Direct vs. Averted						.007*
Ctl: Direct vs. Averted						.81
NVT x Condition	.69	.38	.73	.92	.27	.12
Dynamic gaze condition x outcome	.17	.03*	.19	.94	.05*	.69
ASD: Toward vs. Away		.22			.68	
No ASD: Toward vs. Away		.19			.04*	
Ctl: Toward vs. Away		.004*			<.001*	
NVT x Condition	.76	.75	.96	.67	.71	.49
Dynamic gaze condition x outcome “early ASD group”	.006*	.27	.03*	.92	.006*	.38
Early ASD: Toward vs. Away	.92		.92		.88	
No ASD: Toward vs. Away	.19		.02*		.04*	
Ctl: Toward vs. Away	.01*		.001*		<.001*	
NVT x Condition	.17	.98	.38	.79	.24	.47
(C) Face condition x outcome	.45	.22	.38	.18	.22	.04
ASD: Face vs. Noise						.06*
No ASD: Face vs. Noise						.41
Ctl: Face vs. Noise						.29
NVT x Condition	.51	.69	.56	.34	.70	.89

In the detailed statistical analysis of each face contrast (static gaze, dynamic gaze, face vs. noise), the first set of comparisons focused on potential differences between infants at-risk for autism and the low-risk control group (A). The data were analysed using GLM, with condition as a repeated-measures factor and risk group varying between-subjects, with initial age as a covariate. A similar approach was used for the second set of comparisons, focusing on outcomes at 36-months (B). The latter analysis covaryed both baseline age and nonverbal T-score (NVT) at 36 months. Greenhouse-Geisser corrected p-values are presented where significant group x condition interactions were evident, results from separate analyses by group are presented, to assist in their interpretation. Otherwise, cells are displayed in grey.

Table S2. Participant Characteristics

Visit	Measure	A. Control		B. At-Risk		C. At-Risk: specific profiles	
			Combined	At-Risk no ASD	At-risk ASD	Early ASD*	No ASD-Other concerns**
		mean (SD) *n*	mean (SD) *n*	Mean (SD) *n*	mean (SD) *n*	mean (SD) *n*	mean (SD) *n*
6-10 months	Age at visit (months)	7.4 (1.2) *50*	7.3 (1.2) *54*	7.2 (1.2) *37*	7.5 (1.2) 17	8.0 (1.2) *10*	7.3 (1.1) *12*
	Mullen ELC SS	104.4 (11.3) *50*	94.0 (12.8) *53*	94.9 (10.6) *37*	92.1 (17.3) *16*	91.5 (20.3) *10*	92.8 (8.1) *12*
	Mullen NVT score	56.2 (7.1) *50*	51.5 (8.4) *53*	52.2 (7.8) *37*	49.9 (9.8) *16*	51.0 (11.7) *10*	51.3 (6.3) *12*
	VABS ABC SS	101.8 (13.7) *49*	92.1 (14.8) *53*	93.1 (15.5) *36*	90.0 (13.4) *17*	90.0 (15.3) *10*	87.6 (9.0) *12*
24-months	Age at visit (months)	23.9 (0.7) *47*	23.9 (1.2) *52*	23.9 (1.2) *36*	24.0 (1.0) *16*	24.0 (0.8) *10*	23.8 (1.1) *12*
	Mullen ELC SS	116.0 (14.0) *42*	102.3 (19.8) *52*	104.3 (17.1) *36*	97.8 (24.7) *16*	94.9 (24.0) *10*	102.0 (16.8) *12*
	Mullen NVT score	56.9 (8.8) *43*	51.6 (9.7) *52*	52.5 (8.8) *36*	49.4 (11.3) *16*	49.1 (10.4) *10*	50.2 (10.5) *12*
	VABS ABC SS	108.2 (12.0) *47*	101.5 (10.6) *52*	102.1 (9.6) *36*	100.0 (12.8) *16*	99.2 (10.7) *10*	99.3 (8.8) *12*
	ADOS Communication		2.1 (1.6) *52*	1.6 (1.3) *36*	3.2 (1.8) *16*	4.2 (1.4) *10*	2.3 (1.3) *12*
	ADOS Social		4.3 (3.0) *52*	3.3 (2.5) *36*	6.6 (2.9) *16*	7.9 (2.2) *10*	3.8 (1.6) *12*
	ADOS Total		6.4 (4.3) *52*	4.9 (3.4) *36*	9.8 (4.3) *16*	12.1 (3.3) *10*	6.0 (2.1) *12*
36-months	Age at visit (months)	38.2 (3.1) *48*	37.7 (3.0) *53*	37.6 (3.4) *36*	37.8 (2.1) *17*	37.1 (1.7) *10*	36.7 (1.8) *12*
	Mullen ELC SS	115.8 (16.3) *48*	105.4 (21.5) *52*	110.1 (15.9) *36*	94.8 (28.5) *16*	93.9 (25.8) *9*	103.4 (19.0) *12*
	Mullen NV T-score	57.8 (9.9) *48*	52.6 (13.0) *52*	55.8 (10.3) *36*	45.3 (15.8) *16*	46.9 (13.7) *9*	53.2 (12.1) *12*
	VABS ABC SS	106.4 (9.1) *48*	96.4 (12.2) *53*	99.4 (9.7) *36*	90.1 (14.6) *17*	88.4 (15.2) *10*	95.7 (10.8) *12*
	ADOS Communication	2.5 (1.5) *48*	3.3 (2.2) *53*	2.9 (1.9) *36*	4.2 (2.5) *17*	5.1 (2.6) *10*	4.8 (1.9) *12*
	ADOS Social	3.2 (3.1) *48*	4.9 (3.5) *53*	3.8 (3.2) *36*	7.4 (2.7) *17*	8.2 (2.8) *10*	7.3 (2.6) *12*
	ADOS Total	5.6 (4.3) *48*	8.3 (5.3) *53*	6.7 (4.8) *36*	11.7 (4.7) *17*	13.3 (5.0) *10*	12.1 (4.1) *12*
	ADI Social	--	4.5 (5.3) *52*	2.2 (3.2) *36*	9.8 (5.5) *16*	9.6 (4.7) *10*	3.4 (4.9) *12*
	ADI Communication	--	4.4 (4.8) *52*	2.7 (3.5) *36*	8.4 (5.1) *16*	8.6 (4.7) *10*	3.6 (5.5) *12*
	ADI Repetitive	--	1.6 (2.0) *52*	0.69 (1.1) *36*	3.6 (2.2) *16*	3.5 (2.2) *10*	1.1 (1.3) *12*

* Above ASD cut-off at 24m ADOS ** Not meeting threshold for ASD diagnosis

Table S3. Average Number of Trials Produced in Each Condition, Average Number of Valid Trials after Artefact Rejection, and Number of Infants Included in Subsequent Analysis

Contrast		Control		At-risk					
				Combined		No ASD		ASD	
		mean	*sd*	mean	*sd*	mean	*sd*	mean	*sd*
Static Gaze									
Direct	Trials	35.0	*8.7*	35.3	*8.8*	34.8	*9.2*	36.7	*8.8*
	Valid	20.8	*5.9*	22.6	*7.3*	22.1	*6.3*	23.1	*9.9*
Averted	Trials	35.0	*8.8*	35.5	*8.7*	34.9	*9.1*	37.1	*8.5*
	Valid	20.7	*6.5*	23.2	*6.8*	22.6	*5.1*	23.9	*10.2*
n		*32*		*32*		*22*		*10*	
Gaze shift									
Toward	Trials	128.6	*37.1*	127.5	*38.5*	129.1	*37.1*	123.6	*43.5*
	Valid	58.7	*29.5*	63.0	*33.3*	63.6	*30.9*	59.2	*38.1*
Away	Trials	129.1	*38.3*	125.9	*38.6*	127.4	*37.1*	122.2	*45.1*
	Valid	59.8	*30.5*	63.6	*33.5*	64.4	*30.7*	59.2	*39.1*
n		*45*		*50*		*33*		*16*	
Face vs. Noise									
Face	Trials	69.0	*17.2*	67.1	*17.3*	66.5	*18.2*	68.4	*16.5*
	Valid	39.4	*13.2*	39.4	*17.4*	39.4	*15.1*	37.9	*21.7*
Noise	Trials	46.8	*10.4*	45.9	*13.1*	45.3	*12.9*	47.9	*13.8*
	Valid	26.5	*8.7*	26.7	*9.9*	27.2	*9.6*	25.5	*11.1*
n		*35*		*41*		*27*		*13*	

Supplemental Experimental Procedure

Confirmation of Risk Status in the Older Sibling

At the time of enrolment in the British Autism Study of Infant Siblings (BASIS), none of the participating infants had been diagnosed with any major medical or developmental condition. Infants at-risk all had an older sibling (hereafter, proband) with a community clinical diagnosis of an autism spectrum disorders (ASD; or in 4 cases, a half-sibling), and in 3 cases 2 probands with an ASD. 45 probands were male, 9 were female. Proband diagnosis was confirmed by two expert clinicians (PB, TC) based on information using the Development and Wellbeing Assessment (DAWBA)[1] and the parent-report Social Communication Questionnaire (SCQ)[2]. Most probands met criteria for ASD on both the DAWBA and SCQ (n = 44). While a small number scored below threshold on the SCQ (n = 4) no exclusions were made, due to meeting threshold on the DAWBA and expert opinion. For 2 probands, data were only available for either the DAWBA (n = 1) or the SCQ (n=1). For 4 probands, neither measure was available (aside from parent-confirmed local clinical ASD diagnosis at intake). Parent-reported family medical histories were examined for significant medical conditions in the proband or extended family members, with no exclusions made on this basis.

Infants in the low-risk group were recruited from a volunteer database at the Birkbeck Centre for Brain and Cognitive Development. Inclusion criteria included full-term birth (with one exception), normal birth weight, and lack of any ASD within first-degree family members (as confirmed through parent interview regarding family medical history). All low-risk infants had at least one older-sibling (in 3 cases, only half-sibling/s). 28 of the older siblings were male, 22 were female. Screening for possible ASD in these older siblings was undertaken using the SCQ, with no child scoring above instrument cut-off for ASD (>15) (one score was missing).

Participants' Background Characterisation Measures

Two measures of general developmental level were obtained for the infants and toddlers at each visit. The Mullen Scales of Early Learning (MSEL)[3] is a direct assessment of verbal and non-verbal abilities appropriate for children from birth to 6 years. Scores across four domains – Visual Reception, Fine Motor, Receptive Language, and Expressive Language – are combined to yield an overall Early Learning Composite (ELC; mean = 100, *sd* = 15). Gross motor skills are also assessed but do not contribute to the ELC. An estimate of non-verbal developmental ability (NVT-score) was computed by averaging the T scores (mean = 50, *sd* = 10) for Visual Reception and Fine Motor subscales. The Vineland Adaptive Behavior Scales (VABS)[4] is a parent-report measure of everyday skills in the domains of Communication, Daily Living Skills, Social Interaction, and Motor Skills. These combine to yield an Adaptive Behaviour Composite (ABC; mean = 100, *sd* = 15).

These developmental assessments were undertaken at each of the visits, when infants were aged 6- to 10-months and again around the second and third birthday. Independent research teams conducted assessments in the first two and final two visits. While the MSEL is always administered directly with the child, the VABS has alternative administration formats. The Parent/Caregiver Rating Form (i.e., questionnaire booklet) was used at the 6- to 10-month visit, and the Survey Interview Form was used at the 24-month and 36-month visits. Scores from these measures are presented in Table S2.

Outcome Characterization of the At-Risk and Low-Risk Groups

Alongside the standard measures of cognitive (MSEL) and adaptive (VABS) development taken at each visit, at 24 months (at-risk group only, 50 Module 1, 2 Module 2) and 36 months (both groups; 3 Module 1, 98 Module 2) a semi-structured play assessment, the Autism Diagnostic Observation Schedule (ADOS; Figure 1)[5] was used to assess autism-related social and communication behavioural characteristics. This was augmented at 36 months (at-risk group only) with the parent-report Autism Diagnostic Interview (ADI)[6]. Scores from these assessments are reported in Table S2.

In order to preserve the strongest aspects of our longitudinal design, we sought to separate out the purposive sampling of the two cohorts (at-risk vs. low-risk) and the characterisation of outcomes in the at-risk cohort (ASD vs. non-ASD at 36-months). Details of each group are presented in Table S2. Furthermore, two profiles (subgroups within these categories (Table S2.C)) were specifically of interest to our analysis as described in the main text. These were toddlers whose symptoms appeared early by 24-

month and persisted into 36-months. The second profile was those infants within the at-risk group who were classified as not having ASD but nevertheless exhibiting some form of developmental concern. Ascertainment of these outcomes as well as specific profiles is detailed below.

For the at-risk group consensus ICD-10[7] ASD (including childhood autism, atypical autism, and other pervasive developmental disorder (PDD)) was diagnosed using all available information from all visits by experienced researchers (TC, KH, SC, GP). From the initial group of 53 toddlers assessed at 36-months, 17 (11 boys, 6 girls) met criteria for an ASD diagnosis (32.1%). Given the young age of the children, and in line with the proposed changes to DSM-5, no attempt was made to assign specific sub-categories of PDD/ASD diagnosis. Within the group of toddlers who were classified as having ASD at 36-months, 10 met ADOS cut-off for ASD at 24-months and were therefore considered as exhibiting earlier expression of ASD relative to other toddlers in the At-risk ASD group (Table S2.C). Another subgroup of toddlers from the at-risk group who were classified as not having ASD were considered to still have other developmental concerns (Table S2.C). These were 12 toddlers (22.6%; 3 boys, 9 girls) who either scored above the ADOS or ADI[8] cut-off for ASD or scored <1.5SD on the Mullen ELC or RL and EL subscales but did not meet ICD-10 criteria for an ASD (9 scored > ADOS cut-off, 1 > ADOS cut-off and <1.5SD Mullen ELC cut-off, 1 > ADI cut-off, and 1 <1.5SD Mullen ELC cut-off).

Pilot Study

In our previously published pilot ERP study[9], we tested a separate group of infants at-risk for autism and a control group (n = 62). A 64-channel Geodesic sensor net was used to record EEG. The electrical potential was amplified with .1–100 Hz bandpass, digitized at 250-Hz sampling rate. Infants were presented with static faces displaying either direct or averted gaze (one of the three contrasts in the current study), identical to those used in previous studies with typical infants and children with autism. From the whole group, data from 19 infants at-risk and 17 low-risk controls were retained for analysis. These two groups did not differ in their P1 and N290 response components. However, latency of the P400 component differentiated the two groups, with infants at-risk exhibiting slower P400 to direct gaze, compared to controls. This pilot study served to provide an initial demonstration of differences in the neural sensitivity to eye gaze of infants at-risk for autism. A number of methodological features were improved upon from this pilot study, for the current study. First, we measured ERP to gaze shifts toward vs. away from the infant, rather than only to static gaze. Gaze shift stimuli are more realistic and therefore are thought to engage a wider range of social brain mechanisms and could therefore be more sensitive predictors of outcome. Second, the task was adapted to provide a comparison condition with non-face stimuli, to assess whether the observed ERP effects in response to gaze would be attributable to the neural processing of faces (compared to non-face stimuli). Third, several measures were taken to minimise data loss, e.g., more breaks between tasks. Finally, 64-channel Geodesic infant nets were replaced with 128-channel hydrocel nets, using 500-Hz rather than 250-Hz sampling rate, allowing for higher density and better quality recording.

Detailed Procedure

Participants' overall behaviour was initially coded from videotape. Trials were retained only when infants were fixating the centre of the screen at stimulus onset, without any gaze shifts, blinking, or head movements during the 800 ms segment following onset of the face stimulus or gaze shift. Data were then corrected to the baseline. Beyond the EEG data filters used for recording, no further filtering was undertaken at the analysis stage, as this might introduce distortions to the signal (however, figures in the current report present filtered data for demonstration purposes). In our pilot study, we found comparable results using both filtered and unfiltered data, further reinforcing this decision. Artefact rejection was then undertaken by an experienced EEG researcher (EM), through visual inspection of individual trials, with the data from any sensor excluded if they contained artefacts. Rejection procedures followed established norms, including removal of segments affected by head, body or eye movement, and including those segments (identified during the video coding procedure) where the infant displayed gaze shifts or looked away from the screen during stimulus presentation. Missing data from 12 or fewer channels were interpolated. Otherwise the entire trial was rejected. Data were then referenced to the average.

The task was designed to assess three contrasts within the same group of infants: faces (static face vs. visual noise stimuli), static gaze (faces with direct vs. averted gaze), and dynamic gaze shifts

(toward vs. away from the infant). Figure S2 shows different trial groupings corresponding to each contrast. Trial types included in the *face-noise contrast* comprised all of the valid first trials at the beginning of each presentation block, following presentation of a small fixation stimulus. These were either faces (irrespective of the direction of gaze) or noise stimuli. The second *static direct-averted contrast* further separated all first face trials within each block into those with direct gaze and those with averted gaze. The final contrast – *gaze shifts toward-away from the infant* - compared any such subsequent trials after appearance of the initial face within each block.

Because of variable rates of presentation of each stimulus type, a different number of trials were included for each contrast. Across all contrasts, only those infants who produced a minimum of 10 valid trials per condition were included in the analysis of any given condition. The number of infants included in each contrast is presented in Table S3. The average number of trials and valid trials produced by each risk-group/outcome subgroup did not differ (all p n.s.).

Ascertainment and Characteristics of ERPs in Response to Static Gaze, Dynamic Gaze Shifts, and Faces

Characteristic upward or downward deflections from the baseline, frequently labelled P1, N290, P400, are consistently observed across a range of visual tasks in infancy[10]. Although these components are not, in themselves, specific to face or gaze processing, task-dependent manipulations in faces or gaze direction are widely acknowledged to result in this characteristic waveform. As expected from previous studies, visual inspection of the grand average (constructed separately for each condition across the three contrasts) revealed task-dependent ERPs over occipital channel groups. Across all three contrasts undertaken in the current study, these components were clearly observed in overlapping occipito-temporal channels. Channels were selected within this region of interest through visual inspection of the grand average. For each contrast, those occipito-temporal channels demonstrating the characteristic waveform were selected, avoiding any particularly noisy channels. Figure S3 shows the average waveforms specific to the selected channel groups for the three contrasts. As evident in Figure S3, response amplitude to gaze shifts was restricted relative to the two other contrasts. This was expected given that that the ERP in those trials is time-locked to apparent motion of the eyes on the face, without any overall change in the face on which gaze shifts occur. By contrast, ERPs in the two other contrasts are time-locked to the appearance of a stimulus after a small fixation, and thus result in larger amplitude waveforms. Thus, gaze shift ERPs were more akin to an infant Visual Evoked Potential (VEP) paradigm.

Supplemental References

1. Goodman, R., Ford, T., Richards, H., Gatward, R. & Meltzer, H. The Development and Well-Being Assessment: description and initial validation of an integrated assessment of child and adolescent psychopathology. *J Child Psychol Psychiatry* 41, 645-655 (2000).

2. Rutter, M. *Social Communication Questionnaire*. (WPS: Los Angeles, 2003).

3. Mullen, E.M. *Mullen Scales of Early Learning*. 34, (American Guidance Service: 1995).

4. Sparrow, S. *Vineland II: A Revision of the Vineland Adaptive Behavior Scales*. (American Guidance Service: Circle Pines, 2005).

5. Lord, C. *et al.* The autism diagnostic observation schedule-generic: a standard measure of social and communication deficits associated with the spectrum of autism. *J Autism Dev Disord* 30, 205-223 (2000).

6. Lord, C., Rutter, M. & Le Couteur, A. Autism Diagnostic Interview-Revised: a revised version of a diagnostic interview for caregivers of individuals with possible pervasive developmental disorders. *J Autism Dev Disord* 24, 659-685 (1994).

7. World Health Organization, *The ICD-10 classification of mental and behavioural disorders: diagnostic criteria for research*. (World Health Organization: 1993).

8. Risi, S. *et al.* Combining Information From Multiple Sources in the Diagnosis of Autism Spectrum Disorders. *Journal of the American Academy of Child & Adolescent Psychiatry* 45, 1094-1103 (2006).

9. Elsabbagh, M. *et al.* Neural correlates of eye gaze processing in the infant broader autism phenotype. *Biological Psychiatry* 65, 31-38 (2009).

10. De Haan, M. Johnson, M.H. & Halit, H. Development of face-sensitive event-related potentials during infancy: a review. *International Journal of Psychophysiology* 51, 45-58 (2003).
